# Deep learning-based classification of student GPA integrating psychological and family factors in the post-pandemic era

**DOI:** 10.3389/fpsyg.2026.1696610

**Published:** 2026-03-05

**Authors:** Hongrong Zhang, Fang Fang, Yi Wang, Yong Huang, Ya Li

**Affiliations:** School of Medical Information Engineering, Anhui University of Chinese Medicine, Hefei, Anhui, China

**Keywords:** deep learning, family background, GPA, post-pandemic era, psychological evaluation

## Abstract

**Background:**

In the post-pandemic era, college students’ academic performance is influenced by a range of non-cognitive factors, which often reduces the accuracy of conventional Grade Point Average (GPA) prediction models. For this, we developed a deep-learning–based GPA classification framework that integrates family background and psychological evaluation indicators, and empirically revealed the underlying associations among these dimensions.

**Methods:**

Data were collected from 1,692 undergraduates at a Chinese university. The dataset included family background factors such as gender, family economic situation, only-child, and left-behind years, as well as SCL-90 psychological evaluation scores and GPA records. Four deep learning models were evaluated: TabTransformer, DCNv2, AutoInt, and MLP-ResNet. In addition, a lightweight feature-gating mechanism was incorporated to improve feature selection in high-dimensional heterogeneous data. Model performance was evaluated using Accuracy and Area Under the ROC Curve (AUC). Associations among variables were analyzed using Spearman’s rank correlation, *χ*^2^ tests, and t-SNE visualization.

**Results:**

The TabTransformer with the gating mechanism achieved the highest performance among the tested models, with an Accuracy of 0.798 and an AUC of 0.833. GPA was significantly negatively correlated with SCL-90 domains, including depression and anxiety. Additionally, unfavorable family background factors—such as lower family economic status and longer periods of being left behind—were correlated with poorer psychological assessment outcomes.

**Conclusion:**

This study developed a deep-learning framework using family background and psychological evaluation factors to classify GPA, support academic risk identification, and inform targeted academic assistance and psychological interventions.

## 1 Introduction

In the new normal of higher education during the post-pandemic era, UNESCO (2023) reported that after the historic disruption of the COVID-19 pandemic, most schools are back open worldwide but education is still in recovery assessing the damage done and lessons learned (Harmey and Moss, 2023; Chatzipanagiotou and Katsarou, 2023). Fully understanding and promoting students’ academic success has become a significant global concern. GPA, as a widely used indicator of academic achievement, can help identify students at potential academic risk and provide proactive support (Guanin-Fajardo et al., 2024; Jin, 2023). Traditional GPA prediction models based on admission scores have demonstrated reasonable predictive performance across various educational settings; however, in the more complex and dynamic learning environment of the post-pandemic era, their applicability and accuracy remain limited.

Previous studies have indicated that non-cognitive factors play a significant role in explaining academic achievement (Privado et al., 2024). Non-cognitive factors refer to a set of psychological and social variables that influence learning and behavior beyond traditional cognitive abilities such as intelligence or test scores, including motivation, emotions, psychological well-being, interpersonal relationships, and family environment (Liu et al., 2025; Wong and Chapman, 2024). Although these factors do not directly measure knowledge proficiency, they substantially affect students’ learning processes and sustained engagement. Notably, the COVID-19 pandemic, as a major global life event, has profoundly altered students’ learning patterns (e.g., prolonged online learning), social interactions, and psychological states, rendering the influence of non-cognitive factors more complex and critical (Cerutti et al., 2024; Lee, 2024; Zhao and Akhter, 2023). Among these factors, students’ psychological well-being has become particularly prominent in the post-pandemic era. Recent studies have confirmed that mental health issues often manifest as ‘long-lasting legacy problems’ rather than disappearing immediately; even after restrictions were lifted, students have continued to exhibit interpersonal sensitivity and psychological distress resulting from prolonged social deprivation (Liu et al., 2025; Cai et al., 2025; Wang et al., 2022). These enduring pandemic experiences have exacerbated anxiety, depression, and feelings of loneliness among adolescents, which directly contribute to academic burnout and underachievement (Liu et al., 2024). Simultaneously, the impact of family background has also become more intricate; for example, economic shocks during the pandemic may have intensified resource inequalities among students from different family backgrounds, and prolonged home-based learning may amplify the unique effects of left-behind experiences or only-child status on students’ socio-emotional development (Bertoletti et al., 2023; Yajie et al., 2023). This suggests that family factors may further exacerbate the vicious cycle of academic underachievement, with impacts potentially extending well beyond the immediate disruptions of the pandemic (Deep et al., 2025). Therefore, current academic prediction research urgently requires establish a new paradigm that can comprehensively integrate non-cognitive factors and respond to post-pandemic educational challenges.

Concurrently, while numerous studies have employed machine learning approaches to analyze academic achievement, they remain subject to specific limitations. First, psychological variables are frequently oversimplified or treated merely as auxiliary features, lacking systematic integration within the model. Second, existing applications rarely prioritize interpreting the underlying relational structure between educational and psychological variables (Guanin-Fajardo et al., 2024). To address these complexities, Deep learning models have attracted considerable attention due to their powerful automatic feature extraction capabilities and their advantages in modeling high-dimensional, nonlinear and heterogeneous data. Compared with traditional methods that rely on manual feature selection or predefined variable interactions, deep learning can automatically learn latent structures from complex datasets with minimal prior knowledge, demonstrating stronger generalization and expressive capabilities (Lagares Rodríguez et al., 2025). For instance, Junejo et al. (2025) successfully employed neural network-based models on the Open University Learning Analytics dataset to accurately classify multi-class academic performance and identify at-risk students. This suggests that such computational approaches are well-suited for disentangling the intricate relationships among student psychology, family background, and academic outcomes.

**Figure 1:**
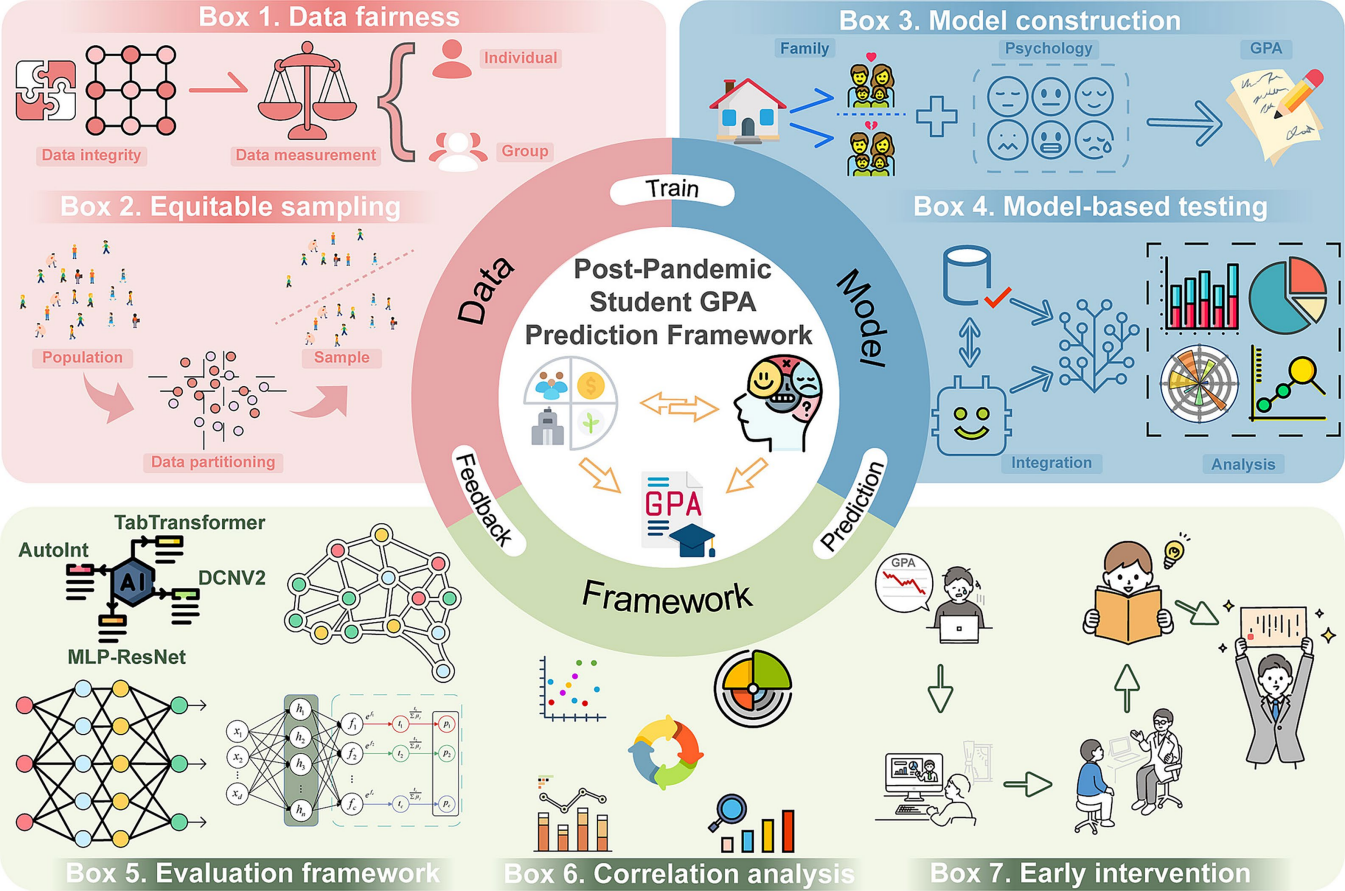
Workflow of this study.

Based on this background, this study proposes the following research questions. First, in the post-pandemic era, what is the association between the psychological status of students and their GPA? Second, are family background factors, such as family economic status, being an only child, and the duration of being left behind, related to student psychological vulnerability? Finally, do family background factors and psychological vulnerability jointly contribute to differences in student GPA? Therefore, this study aims to develop a deep learning and feature-selection-based framework for classifying GPA levels of students in the post-pandemic era. The framework is grounded in real-world data collected from 1,692 undergraduates enrolled in 2023 at a Chinese university, taking into account family background factors such as gender, family economic situation, only-child and left-behind years, while integrating psychological evaluation scores from the Symptom Checklist-90 (SCL-90) scale. Four deep learning models—including TabTransformer, DCNv2, AutoInt, and MLP-ResNet—were employed, and a lightweight feature-gating mechanism was introduced to enhance the prediction of student GPA levels. Furthermore, the framework integrates multidimensional statistical analyses to systematically explore the complex associations among students’ family background, psychological evaluation, and academic performance (Figure 1). This framework not only facilitates identification of academic risk and personalized intervention for students in the post-pandemic era but also provides a valuable reference for research in educational data mining.

## 2 Materials and methods

### 2.1 Data acquisition

The dataset used in this study was drawn from real-world data at a Chinese university, encompassing 1,692 undergraduates enrolled in 2023. The dataset systematically integrates multidimensional individual information collected from three distinct university systems. Academic performance for the current academic year, measured by GPA, was retrieved directly from the Academic Affairs System. Family background data, including gender, family economic situation, only-child status, and years of left-behind experience, were obtained through questionnaire surveys administered by the Student Affairs System. Additionally, the dataset incorporates psychological assessment data, specifically scores from the SCL-90 scale, which were derived from survey results provided by the Mental Health Education Center.

The SCL-90 is a widely used self-report measure of psychological health, comprising 10 psychological dimensions: somatization, obsessive compulsive symptoms, interpersonal sensitivity, depression, anxiety, hostility, phobic anxiety, paranoid ideation, psychoticism, and additional items, totaling 90 items. Participants rated their psychological and physical states over a recent period on a five-point scale ranging from 1 (none) to 5 (severe) (Li and Zhang, 2023). The scale scores were used to reflect the severity of symptoms across each psychological dimension; definitions of the dimensions and item distribution are detailed in Table 1. Data integration was performed using unique student identification numbers as the primary key to ensure accurate matching across these three sources. To strictly protect student privacy, a rigorous anonymization protocol was implemented. Immediately after data matching was completed, all personal identifiers, such as names and student IDs, were permanently removed. Consequently, the final dataset utilized for analysis and modeling was fully anonymized and devoid of any personally identifiable information. The study strictly adhered to ethical guidelines and was approved by the university’s ethics committee.

**Table 1:** Overview of family background, psychological dimensions, and GPA categories.

Category	Item	Category
GPA	GPA Level	3.67–4.0/2.67–3.67/0–2.67
Family background	Gender	Female/male
	Family economic situation	Rural-poor/rural-avg/urban-poor/urban-avg
	Left-behind years	0–16
	Only child	Only/non-only
Psychological (SCL-90)	Somatization	1.0–5.0
	Obsessive compulsive symptoms	1.0–5.0
	Interpersonal sensitivity	1.0–5.0
	Depression	1.0–5.0
	Anxiety	1.0–5.0
	Hostility	1.0–5.0
	Phobic Anxiety	1.0–5.0
	Paranoid ideation	1.0–5.0
	Psychoticism	1.0–5.0
	Additional Items	1.0–5.0

### 2.2 Data filtering and preprocessing

During the data preprocessing stage, we conducted rigorous quality control on the raw data. To ensure the seriousness of participants’ responses, we excluded SCL-90 questionnaires completed in less than 200 s, thereby removing potentially inattentive or superficial responses. Additionally, we screened for potentially invalid responses, such as cases where participants selected the same option for 30 consecutive items, and removed them to further ensure data validity and reliability (Zhang et al., 2024). Moreover, to optimize the feature structure, we excluded Additional Items category due to its ill-defined nature and utilized the remaining nine-dimension scores of the SCL-90 as input features for the model. Following data quality control, we standardized and encoded the data according to variable types.

First, for the primary academic performance indicator GPA, we followed common GPA grading standards and considered the sample distribution. Students with GPAs of 3.67–4.0 were classified as “Excellent” (coded as 0), GPAs of 2.67–3.67 as “Good” (coded as 1), and GPAs below 2.67 as “Average” (coded as 2). Second, for the family economic situation variable, “Rural Poor” was coded as 0, “Rural Average” as 1, “Urban Poor” as 2, and “Urban Average” as 3. For the only-child variable, non-only children were coded as 0 and only children as 1; for gender, females were coded as 0 and males as 1. The duration of being left behind was measured in years, ranging from 0 to 16, and expressed as integers. After completing data encoding and standardization, we paired and integrated students’ SCL-90 scores with their GPA and all relevant variables to facilitate subsequent analyses.

Furthermore, the dataset was split into training, validation, and test sets in a 6:2:2 ratio, using stratified sampling to ensure that the distribution of GPA categories remained consistent across subsets.

### 2.3 Model training and validation

To systematically evaluate and compare the performance of different deep learning tabular models on the multi-class GPA prediction task, four methods were selected: TabTransformer, DCNv2, AutoInt, and MLP-ResNet. Each model was adapted and optimized according to its structural characteristics.

### 2.4 TabTransformer

The TabTransformer model requires categorical and numerical features to be input separately. Categorical features, such as gender and economic status, were encoded as integers and fed into the embedding layer, while continuous features, including SCL-90 domain scores, were standardized to zero mean and unit variance and input as floating-point values. Encoded GPA levels served as target labels for classification training and evaluation. All input features and labels were batched using PyTorch’ s TensorDataset and DataLoader.

The TabTransformer consisted of two Transformer encoder layers, each with four attention heads and a dropout rate of 0.4. The model was optimized using Adam with a learning rate of 1e-3 and a weight decay of 1e-4, trained for up to 1,000 epochs with an early stopping patience of 40 epochs. During training, the model operated in training mode in each epoch, with batches of categorical features, continuous features, and GPA labels loaded from the DataLoader. Categorical features were processed through the embedding layer and Transformer encoder, then concatenated with continuous features and passed to the classification layer to produce GPA prediction probabilities. Cross-entropy loss was used to compute the loss, with gradients cleared, backpropagated, and parameters updated at each step. After each epoch, validation loss was calculated for early stopping. Training terminates early if validation loss fails to improve for 40 consecutive epochs, with historical optimal parameters restored to prevent overfitting.

### 2.5 DCNv2

The DCNv2 model comprised three cross-network layers and three deep-network layers, with each layer of the deep branch having a width of 128 and a dropout rate of 0.2. Model parameters were optimized using Adam with a learning rate of 5e-4 and weight decay of 5e-4, trained for up to 1,000 epochs with an early stopping patience of 40 epochs.

During training, input feature vectors were fed simultaneously into the cross-network and deep-network branches. The cross-network captured explicit high-order feature interactions, while the deep network learned more implicit feature patterns. The outputs of the two parallel networks were concatenated and passed through a final linear layer to produce GPA class probabilities. Cross-entropy loss was used, following standard procedures for gradient clearing, backpropagation, and parameter updating. After each epoch, validation loss was computed to monitor model convergence. Training terminates early if validation loss fails to improve for 40 consecutive epochs, with optimal-performing model weights loaded as the final model to ensure generalization capability.

### 2.6 AutoInt

To explicitly model high-order feature interactions, the AutoInt model was introduced. Prior to input, features were processed according to type: continuous features, including SCL-90 domain scores, were standardized to zero mean and unit variance. The core of AutoInt consisted of two interacting layers, each composed of a two-head multi-head self-attention module and a residual connection, designed to capture complex combinatorial relationships among input features. The model outputted GPA class predictions. Training employed the Adam optimizer with a learning rate of 1e-3, integrated with an early stopping mechanism set to a patience of 40 epochs to prevent overfitting.

### 2.7 MLP-ResNet

The MLP-ResNet model was composed of three stacked fully-connected residual blocks, each containing a linear transformation, ReLU activation, and dropout. Inputs and outputs were connected via residual summation, effectively mitigating gradient vanishing and enhancing the representation capability of deep networks. Hidden layers were set to width 64 with a dropout of 0.5 to enhance generalization performance. All input features and labels were batched using PyTorch’ s TensorDataset and DataLoader. During training, the model operated in training mode per epoch, processing batches of concatenated feature vectors and GPA labels through the projection layer and residual blocks to generate categorical GPA predictions. The model was optimized using Adam with a learning rate of 5e-4, weight decay 1e-3, trained for up to 1,000 epochs, and incorporated early stopping with patience of 40 epochs. Standard training procedures began with gradients zeroization, followed by forward propagation, cross-entropy loss computation, backpropagation, and parameters updates, with training loss recorded throughout the process. Training terminates early if validation loss fails to improve for 40 consecutive epochs.

### 2.8 Ablation and integration experiments on gating mechanism

When processing tabular data containing complex information such as personal background and psychological health status, a key challenge is that not all input features contribute equally to the target task (for example, GPA level prediction). In particular, the 90 items of the SCL-90 scale may contain some redundant information or noise unrelated to academic performance. Traditional models may be affected by irrelevant features when processing such data, potentially leading to reduced learning efficiency and increased risk of overfitting.

To address this issue and enhance the models’ dynamic feature filtering capabilities, this study incorporated a lightweight feature gating mechanism into various structured data models. Its advantage lies in dynamically learning feature weights to highlight important information and suppress irrelevant noise, potentially improving the model’s robustness and generalization in high-dimensional heterogeneous feature scenarios (Niu et al., 2024). This module learns a continuous weight coefficient between 0 and 1 for each input feature dimension, enabling adaptive reweighting of the original features to emphasize key features while suppressing irrelevant ones, and potentially enhances the learning effectiveness of the backbone model.

The Gating mechanism is embedded at the input stage of the backbone model as a front-end feature filtering layer, jointly trained with the downstream main model. We individually integrated it into four mainstream model architectures, including TabTransformer, DCNv2, AutoInt and MLP-ResNet, and conducted systematic comparative and ablation experiments.

### 2.9 TabTransformer with gating

The original input of TabTransformer consists of categorical and continuous features. To incorporate the Gating mechanism, categorical and continuous features are first concatenated to form a complete input vector. After weighting by the Gating module, the weighted features are split back into their original structure and fed into the TabTransformer model. This design enables TabTransformer to model feature interactions while adaptively selecting relevant input features.

### 2.10 DCNv2 with gating

DCNv2 takes the fully concatenated feature vector as input, so the Gating module can be directly connected in series before DCNv2, forming a sequential structure with a front-end feature-weighting layer and the backbone network. During training, the Gating parameters are optimized synchronously with DCNv2, collaboratively achieving feature selection and high-order feature interaction modeling.

### 2.11 AutoInt with gating

To properly integrate the Gating mechanism with the AutoInt model, all categorical features are first converted into vectors via embedding layers and concatenated with continuous features to form a unified feature representation vector. Subsequently, the Gating module applies element-wise weighting to this consolidated vector for dynamic feature selection. Finally, the weighted vector is fed into AutoInt’s self-attention network for interaction learning.

### 2.12 MLP-ResNet with gating

Similar to DCNv2, MLP-ResNet takes full feature vectors as input, thus also permitting serial integration where the Gating module serves as a front-end feature-weighting layer. This integration endows MLP-ResNet with superior feature filtering and prominent robustness while preserving its original modeling capability.

Additionally, for all models with integrated Gating, the training procedure, including loss function, batch size, and optimizer remains identical to the original models, ensuring comparability and reliability of the ablation experiments.

### 2.13 Model testing

To ensure objective and rigorous evaluation and comparation of all models’ final performance on the GPA level prediction task, this study conducted a unified evaluation procedure for all trained models on the test set. The evaluation included four baseline models—TabTransformer, DCNv2, AutoInt, and MLP-ResNet—as well as their ablation variants integrated with the Gating module. Considering the potential class imbalance in the GPA categories, a set of classification metrics based on macro-averaging was adopted to ensure that each class received equal attention. The core evaluation metrics comprised Accuracy, Macro-Precision, Macro-Recall, Macro-F1 Score, and the Area Under the ROC Curve (AUC).

Accuracy represents the proportion of correctly predicted samples among all samples and serves as an intuitive indicator of overall performance (Equation 1). Macro-Precision measures the proportion of samples predicted as positive that are actually positive, averaged across all classes (Equation 2). Macro-Recall measures the proportion of actual positive samples that are correctly predicted by the model, averaged across all classes (Equation 3). Macro-F1 is the harmonic mean of macro-Precision and macro-Recall and provides a balanced assessment of the model’s precision and recall performance (Equation 4). In multi-class classification tasks, the study adopts a One-vs-One (OVO) strategy, where the AUC is calculated for every pair of classes and then averaged across all pairs, providing an overall assessment of the model’s discriminative ability under varying thresholds (Equation 5).


$$\text{Accuracy}=\frac{\sum_{k=1}^{K}TP_{k}}{N}\;$$ (1)

$$\text{Macro}-\text{Precision}=\frac{1}{K}\sum_{k=1}^{K}\frac{TP_{k}}{TP_{k}+FP_{k}}\;$$ (2)

$$\text{Macro}-\text{Recall}=\frac{1}{K}\sum_{k=1}^{K}\frac{TP_{k}}{TP_{k}+FN_{k}}\;$$ (3)

$$\text{Macro}-F1=\frac{1}{K}\sum_{k=1}^{K}(\frac{2\times {\text{Precision}}_{k}\times {\text{Recall}}_{k}}{{\text{Precision}}_{k}+{\text{Recall}}_{k}})\;$$ (4)

$$\mathrm{AUC}=\frac{2}{K(K-1)}\sum_{i=1}^{K-1}\sum_{j=i+1}^{K}{\mathrm{AUC}}_{i,j}\;$$ (5)

In these formulas, *K* denotes the number of classes, *TP_k_* denotes the true positives for class *k*, *N* denotes the total number of samples, *FP_k_* denotes the false positives for class *k*, *FN_k_* denotes the false negatives for class *k*, and *AUC_i,j_* denotes the area under the ROC curve between class *i* and class *j*.

All experimental results were visualized using the matplotlib library in Python to provide an intuitive comparison of the performance across different models.

### 2.14 Dimensionality reduction and clustering analysis

To comprehensively investigate the relationships among students’ GPA levels, SCL-90 scores, and family background factors including gender, family economic situation, only-child, and left-behind years, as well as to reveal the underlying sample distribution in high-dimensional data, this study employed a strategy combining statistical tests with nonlinear dimensionality reduction and visualization to analyze the student dataset. According to the SCL-90 standard, the continuous total SCL-90 scores were categorized into normal (<160), mild abnormal (160–250), and severe abnormal (>250) groups (Chen et al., 2020). To visually represent the intrinsic structure and clustering trends of high-dimensional sample data in two-dimensional space, a two-stage dimensionality reduction strategy combining Principal Component Analysis (PCA) and t-Distributed Stochastic Neighbor Embedding (t-SNE) was employed.

First, PCA was applied to the standardized high-dimensional feature matrix to perform a linear transformation. PCA aims to identify the directions of maximum variance in the data, thereby achieving effective dimensionality reduction while retaining most of the information. The number of principal components was adaptively determined using n_components = 0.9. Subsequently, the PCA-reduced data were input into the t-SNE model for nonlinear embedding, ultimately mapping the data into a two-dimensional visualization space. t-SNE is a manifold learning algorithm particularly effective at preserving local neighborhood structures in high-dimensional space. The key hyperparameters were set as follows: perplexity = 30, learning rate = 200, n_iter = 2000, and early_exaggeration = 20. Based on the resulting two-dimensional coordinates, t-SNE scatter plots were generated. In each plot, the spatial positions of the data points remain unchanged, while the color encodes different categories, such as gender, SCL-90 scores, and GPA levels, allowing an intuitive assessment of whether these variables are associated with the natural clustering patterns of the samples.

Furthermore, to assess the statistical association strength among the categorical variables, chi-squared (*χ*^2^) independence tests were performed pairwise on the six core categorical variables: gender, only-child status, family economic situation, years left behind, SCL-90 grouping, and GPA level. Smaller *p*-values indicate a higher likelihood of a significant association between the two variables.

### 2.15 Correlation analysis between SCL-90 factors and GPA

To further investigate the potential relationships among students’ GPA levels, SCL-90 scores, and family background variables, this study employed Spearman’s rank correlation, a non-parametric statistical method. This method compares the ranks of the variables to effectively measure the strength and direction of monotonic relationships, while remaining robust to variable distribution types and outliers. Therefore, it is suitable for continuous, categorical and skewed data (Qiu et al., 2025).

First, the following student variables were extracted from raw datasets: students’ GPA levels, total SCL-90 scores, years left behind, family economic situation, gender, and only-child status. Pairwise correlation analyses were then conducted for each combination of variables. Spearman’s correlation coefficients were calculated to quantitatively reflect the strength and direction of the associations, and corresponding *p*-values were computed to assess statistical significance. Additionally, a more detailed analysis was performed to examine the relationships between the nine SCL-90 psychological health factors and GPA levels. The score for each factor was calculated as the mean of its constituent items. To identify abnormal distributions of students’ psychological health, factor scores exceeding the normative reference values (Table 2) were considered abnormal.

**Table 2:** Normative means and standard deviations for SCL-90 factors.

Factor	Mean ± SD	Factor	Mean ± SD	Factor	Mean ± SD
Somatization	1.37 ± 0.48	Hostility	1.46 ± 0.55	Interpersonal sensitivity	1.65 ± 0.61
Obsessive compulsive symptoms	1.62 ± 0.58	Phobic anxiety	1.23 ± 0.41	Paranoid ideation	1.43 ± 0.57
Depression	1.50 ± 0.59	Psychoticism	1.29 ± 0.42	Anxiety	1.39 ± 0.43

All correlation analyses and visualizations were conducted using Python libraries including scipy, seaborn, and matplotlib. *p*-value < 0.05 was set as the threshold for statistical significance.

## 3 Results

### 3.1 Data screening

**Figure 2:**
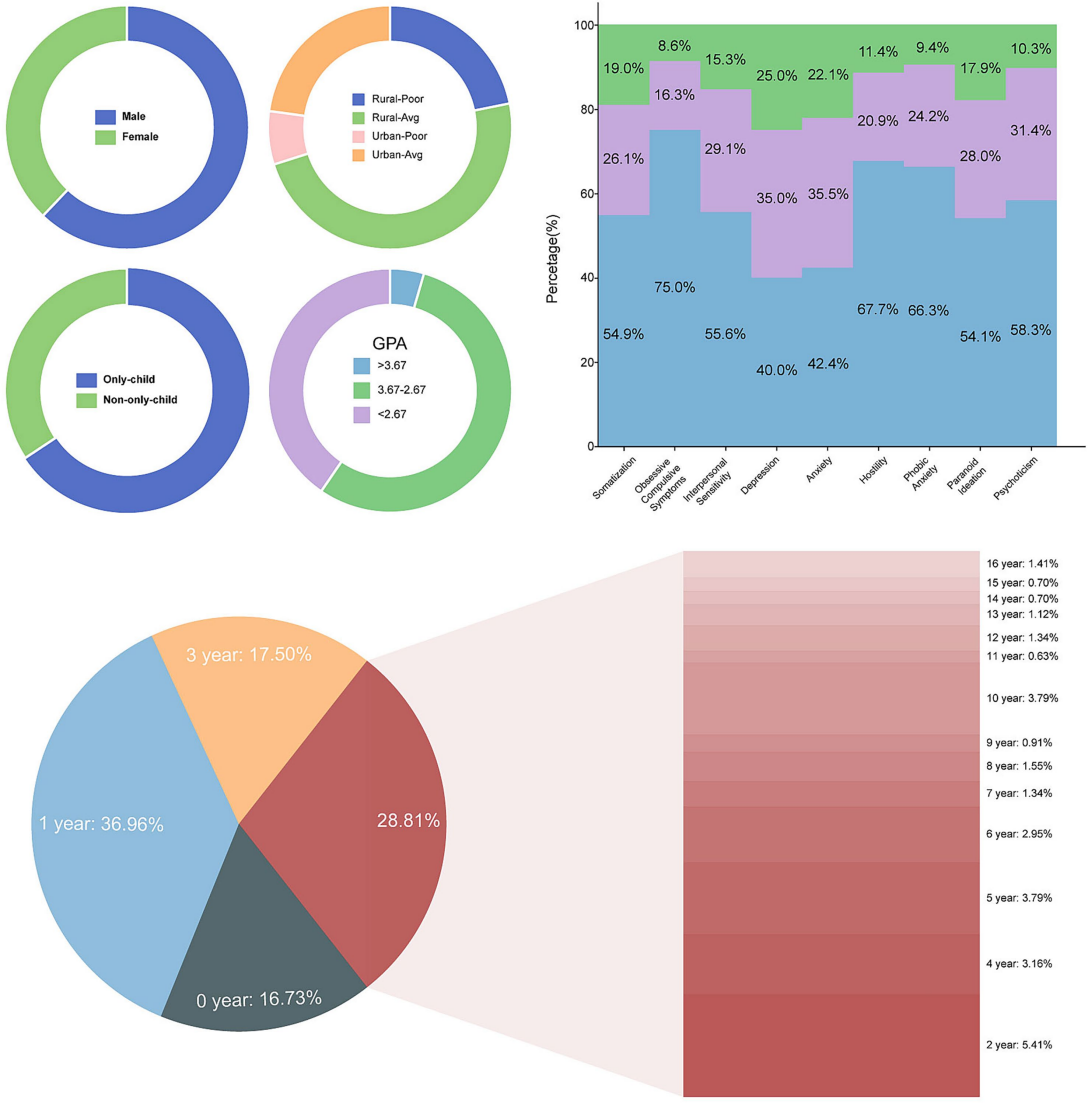
Distribution of family background factors, GPA, and SCL-90 psychological health characteristics in the study sample.

To ensure the validity and reliability of data for subsequent modeling analysis while mitigating potential bias from invalid responses, we implemented rigorous screening and preprocessing of the raw dataset. After these procedures, 57 records were removed, yielding 1,635 valid undergraduate questionnaires for analysis. Figure 2 presents the distributions of sex, family economic situation, only-child, left-behind years, SCL-90 scores, and GPA levels. The sex ratio was approximately balanced, with slightly more females than males. More than half of the students were only children. Family economic situations were diverse: The proportion of students in the Rural-Average group was the largest, followed by Urban-Average, whereas the combined proportions of Rural-Poor and Urban-Poor were lower. Regarding years spent as a left-behind child, 16.73% of students had no left-behind experience (0 years). Among those with such experience, 1 year was most common (36.96%), followed by 3 years (17.50%). The remaining 28.81% were distributed across other durations, with notable proportions at 2 years (5.41%), 5 years (3.79%), and 10 years (3.79%). Detailed distributions are shown in Figure 2.

To assess overall psychological health, we examined score distributions for the nine SCL-90 factors and categorized students into three groups according to clinical criteria: normal, mild abnormal, and severe abnormal. Overall, most students fell within the normal range across all nine factors, although some dimensions showed more pronounced symptoms. Among these, Depression and Anxiety were the two most prominent dimensions of concern: 25.0 and 22.1% of students reached a severely abnormal level on these two factors respectively, while another 35.0 and 35.5% showed mild abnormality. This indicates that more than half of the students exhibited some level of depressive or anxious symptomatology.

Additionally, higher proportions of severe symptoms were observed on factors including Somatization (19.0%), Interpersonal Sensitivity (15.3%), and Paranoid Ideation (17.9%). By contrast, the abnormality rate of Obsessive Compulsive Symptoms was the lowest among the factors, with 75.0% of students scoring in the normal range.

### 3.2 Baseline model performance comparison

**Figure 3:**
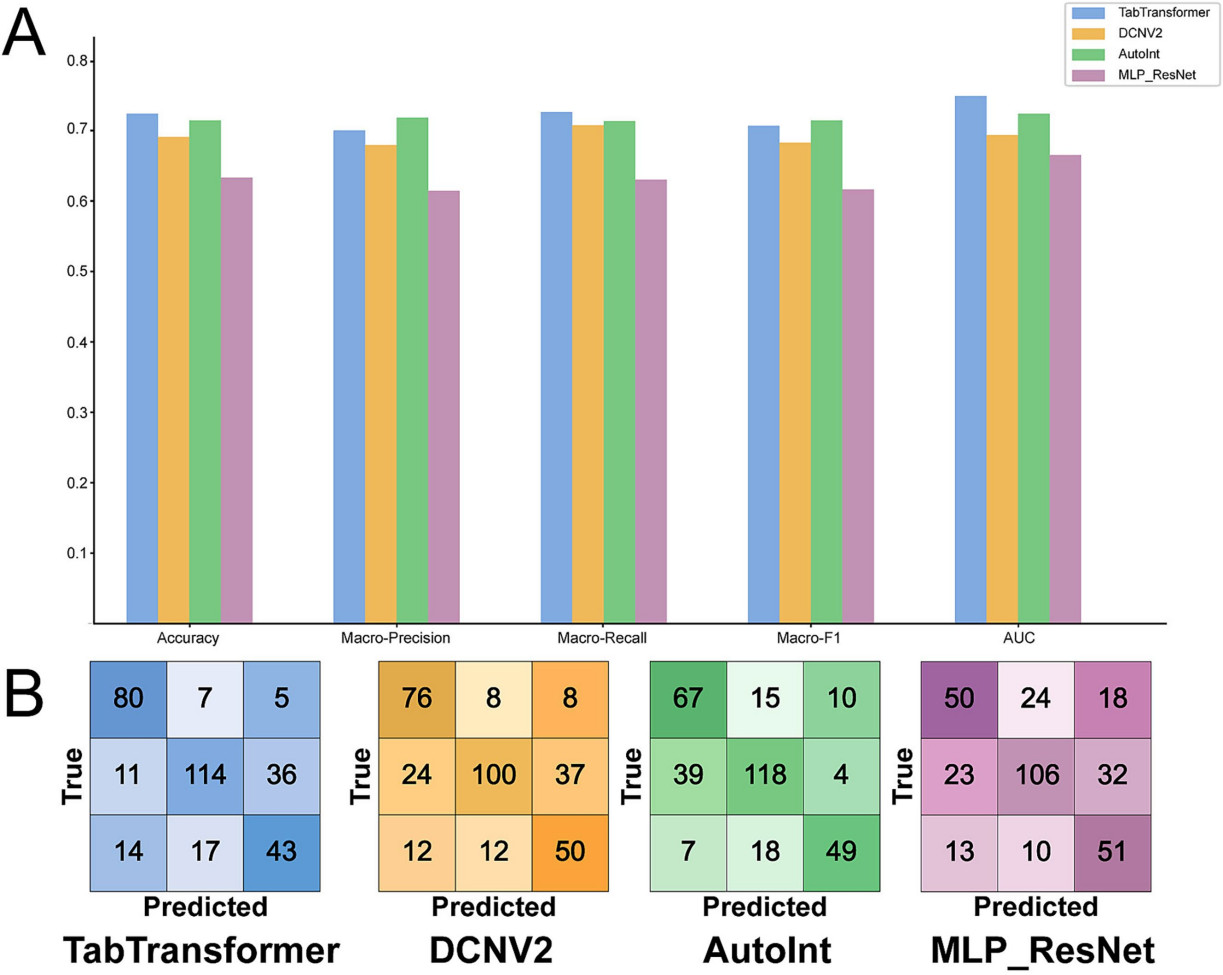
Performance comparison of the four baseline models on the test set. **(A)** Bar chart comparing the four models on five key metrics: Accuracy, macro-precision, macro-recall, macro-F1 score, and AUC. **(B)** The corresponding confusion matrices for each model, detailing the classification results across the three GPA levels.

To systematically evaluate model performance on the GPA-level classification task, we conducted comparative experiments on four deep learning models: TabTransformer, DCNv2, AutoInt, and MLP-ResNet. Test-set results for Accuracy, Macro-Precision, Macro-Recall, Macro-F1, and AUC are summarized in Table 3 and Figure 3A.

**Table 3:** Detailed performance metrics of the four baseline models on the test set for the GPA prediction task.

Model	Accuracy	Macro-precision	Macro-recall	Macro-F1	AUC
TabTransformer	**0.7248**	0.7001	**0.7196**	0.7063	**0.7492**
DCNV2	0.6911	0.6794	0.7076	0.6829	0.6934
AutoInt	0.7156	**0.7174**	0.7078	**0.7126**	0.7264
MLP-ResNet	0.6330	0.6145	0.6304	0.6163	0.6652

Bold values indicate the best model performance scores.

Overall, there were significant differences in the performance of different models. TabTransformer achieved the highest or second-highest scores on all five metrics (Accuracy = 0.7248; Macro-Recall = 0.7196; AUC = 0.7492), indicating the strongest overall performance among the baselines. AutoInt also performed competitively, yielding the highest Macro-Precision (0.7174) and a Macro-F1 of 0.7126. DCNv2 showed mid-range performance, with metrics slightly below those of TabTransformer and AutoInt. MLP-ResNet had the lowest scores among the baselines (Accuracy = 0.6330; AUC = 0.6652), suggesting reduced effectiveness on this task without additional inductive mechanisms for heterogeneous tabular data.

Confusion matrices in Figure 3B indicate that all baseline models performed best at classifying the middle category “Good” (GPA 2.67–3.67), yet struggled to distinguish between “Excellent” (GPA > 3.67) and “Average” (GPA < 2.67) categories, frequently confusing both with “Good.” For example, the best-performing TabTransformer misclassified 36 “Good” cases as “Average” and 14 “Average” cases as “Excellent,” highlighting the residual difficulty of baseline models in handling samples with ambiguous class boundaries.

### 3.3 Ablation results for the gating mechanism

**Figure 4:**
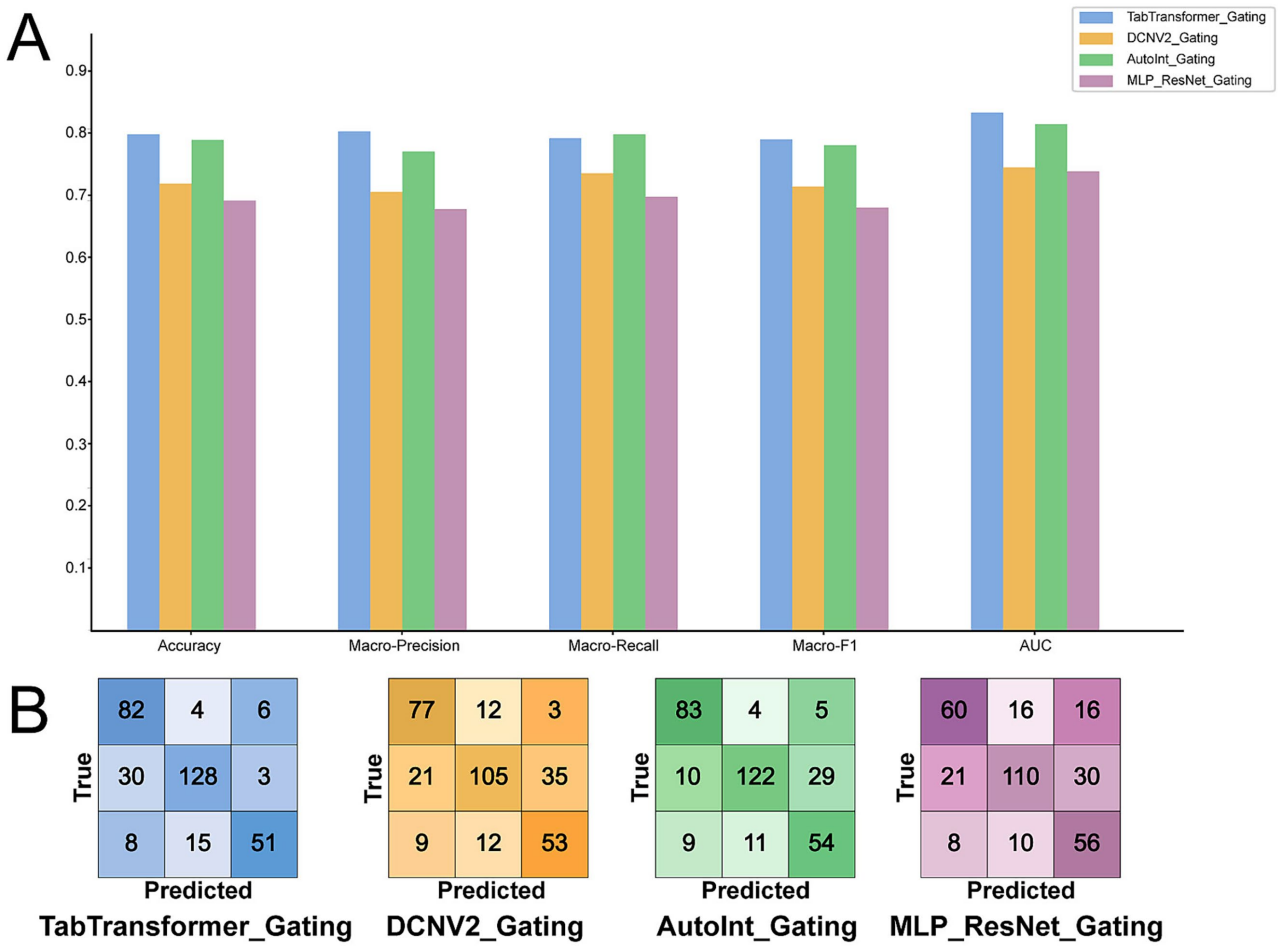
Performance comparison of the four models after integrating the Gating mechanism on the test set. **(A)** Bar chart comparing the four Gating-integrated models on the five key performance metrics. **(B)** The corresponding confusion matrices for each integrated model, reflecting the improvements in classification discrimination after the introduction of the Gating mechanism.

To quantitatively assess the contribution of the Gating mechanism to model performance, we integrated it as a pre-input feature-weighting module into all four baseline models and conducted a controlled ablation study. Detailed post-integration metrics are reported in Table 4, and Figure 4 summarizes both the magnitude of the performance gains and the changes in classification behavior.

**Table 4:** Detailed performance metrics of the four models after integrating the Gating mechanism on the test set for the GPA prediction task.

Model	Accuracy	Macro-precision	Macro-recall	Macro-F1	AUC
TabTransformer + Gating	**0.7982**	**0.8013**	0.7918	**0.7887**	**0.8328**
DCNV2 + Gating	0.7187	0.7053	0.7351	0.7135	0.7446
AutoInt + Gating	0.792	0.7726	**0.7966**	0.7844	0.8171
MLP-ResNet + Gating	0.6911	0.6773	0.6974	0.6800	0.7382

Bold values indicate the best model performance scores.

The experiments indicate that the lightweight Gating module improved the performance of all baselines. As shown in Figure 4A, compared with their unaugmented counterparts, all models augmented with the Gating module achieved consistent increases in Accuracy, Macro-Precision, Macro-Recall, Macro-F1, and AUC. This pattern suggests that the Gating module is not an optimization specifically for a particular model architecture, but rather a performance enhancement strategy with wide applicability.

In particular, TabTransformer augmented with the Gating module yielded the best overall results for GPA prediction. Its Accuracy increased from 0.7248 to 0.7982 (an absolute gain of 0.0734; 7.34 percentage points), and its AUC rose from 0.7492 to 0.8328 (an absolute gain of 0.0836; 8.36 percentage points), exceeding 0.80. AutoInt augmented with the Gating module also showed strong gains, reaching an Accuracy of 0.7920 and improving AUC from 0.7264 to 0.8171. DCNv2 augmented with the Gating module exhibited clear improvements as well. Notably, even MLP-ResNet augmented with the Gating module improved its AUC from 0.6652 to 0.7382—surpassing all other baseline models except the unaugmented TabTransformer (AUC = 0.7492; see Table 3). These findings imply that the Gating module can compensate for limited feature handling in simpler architectures.

Closer inspection of confusion matrices suggests that the gains stem from enhanced class discrimination. Comparing Figure 4B with Figure 3B, the augmented models reduced characteristic error patterns. Relative to the baselines, models with the Gating module improved differentiation between the “Average” (GPA < 2.67) and “Excellent” (GPA > 3.67) categories, which were previously prone to confusion with the “Good” class (2.67–3.67). For example, TabTransformer with the Gating module increased correct identifications in the “Good” class from 114 to 128 and in the “Excellent” class from 80 to 82, with concomitant improvements for “Average.” AutoInt with the Gating module showed a similar trend, increasing correct “Excellent” classifications from 67 to 83 and reducing misclassifications within the “Good” class. DCNv2 and MLP-ResNet with the Gating module also improved recognition of “Average” and “Good” respectively, indicating positive effects across architectures. Overall, the augmented models exhibited fewer tendencies to default to the majority “Good” class and better recognition of minority classes.

Taken together, the ablation results indicate that the Gating module, by dynamically learning feature importance and adaptively weighting inputs, helped suppress noise in high-dimensional data. This allowed the models to focus more on signals relevant to GPA prediction, improving discrimination among easily confused categories and yielding consistent gains in overall performance.

### 3.4 Association analysis and distribution characteristics

**Figure 5:**
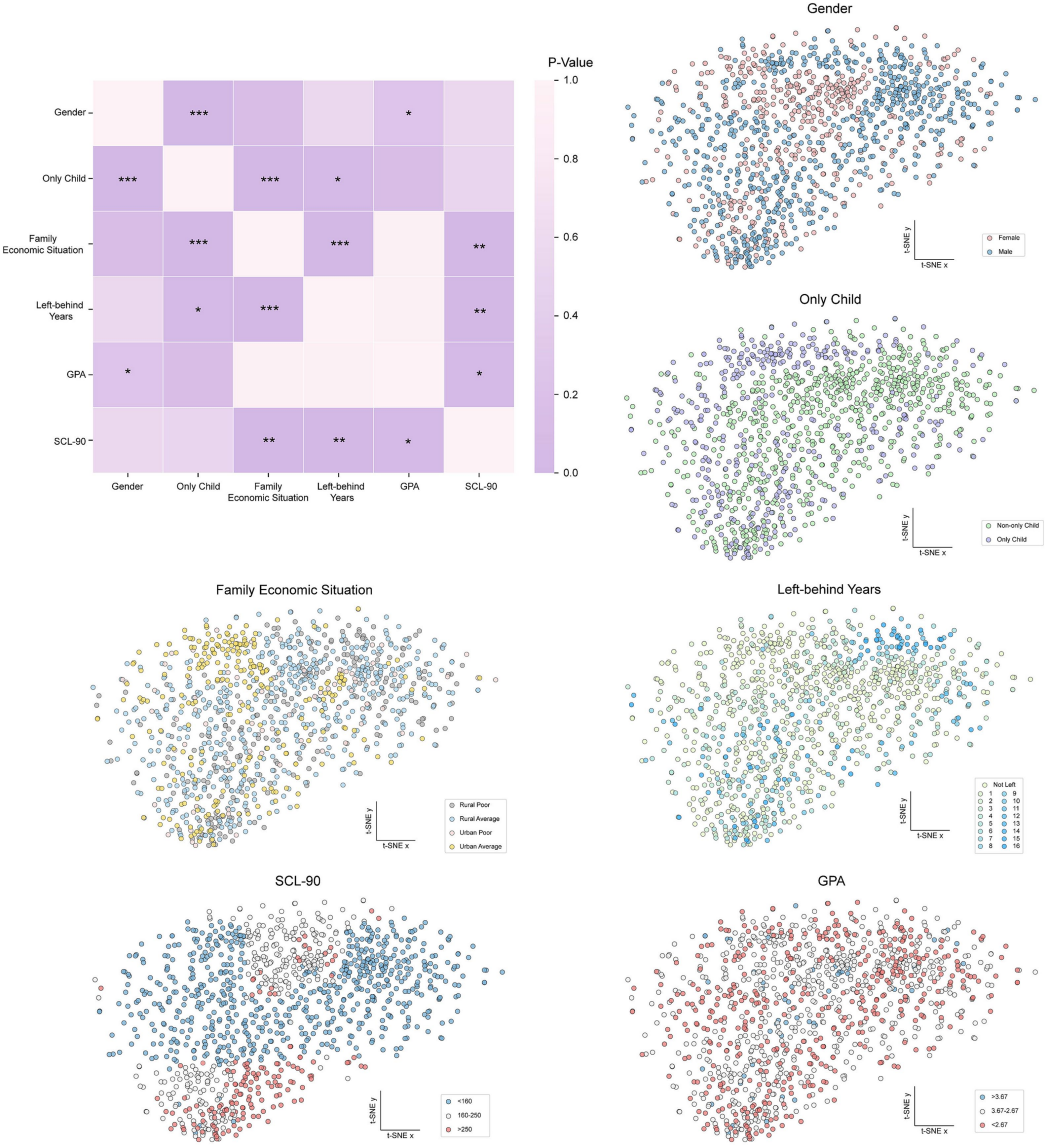
Multivariate correlation analysis and *t*-SNE visualization of family background, psychological health, and GPA. *p < 0.05, **p < 0.01, *** *p* < 0.001.

To explore the associations among students’ family background, psychological factors, and GPA, while examining distribution patterns in the feature space, we combined *χ*^2^ tests of independence with t-SNE dimensionality reduction. The results are shown in Figure 5.

From a statistical perspective, several pairs of variables showed significant associations. Specifically, SCL-90 grouping was significantly associated with family economic situation (*p* < 0.01) and left-behind years (*p* < 0.01), suggesting that economic stress and prolonged left-behind experiences may be potential correlates of students’ psychological health. GPA was also statistically associated with SCL-90 grouping (*p* < 0.05), which suggests a relationship between academic performance and psychological status. Expected internal associations among family background variables were observed as well; for example, family economic situation was significantly associated with both only-child status (*p* < 0.001) and left-behind years (*p* < 0.001).

In addition, the intrinsic distribution analysis suggests that different variables contribute unequally to the partitioning of the sample space. We projected the high-dimensional student feature data into two dimensions using t-SNE to visualize potential clustering tendencies. In each t-SNE plot, every point represents an individual student, and their spatial coordinates remain consistent across the variable-colored subplots. The t-SNE map for gender suggests some spatial separation between male and female students. Although there is substantial overlap, clustering tendencies remain observable. The only-child t-SNE plot suggests partial separability between only-child and non-only-child students, particularly in high-density regions where clustering is apparent. The t-SNE analysis colored by family economic situation suggests differences in the reduced space. Students from rural versus urban backgrounds and from economically average versus poor families tend to occupy different subregions, which may indicate an influence of economic factors on overall feature distribution. The t-SNE map for left-behind years suggests that students with no left-behind experience and those with such experience occupy different regions; students with longer left-behind durations tend to form local clusters in the dimensionality-reduced space. Notably, the t-SNE map for SCL-90 grouping exhibits pronounced stratification within the embedded space, with significant distributional separation between the severe-abnormal and normal groups, which may indicate that psychological health status influences students’ overall feature structure. The GPA t-SNE plot likewise suggests that students of different GPA grades show a certain clustering tendency trend in the reduced-dimensional space. Students with higher and lower GPAs tend to be separated in the two-dimensional embedding space, which may be consistent with a complex association between academic achievement and multi-dimensional background variables.

### 3.5 Correlation analysis results

**Figure 6:**
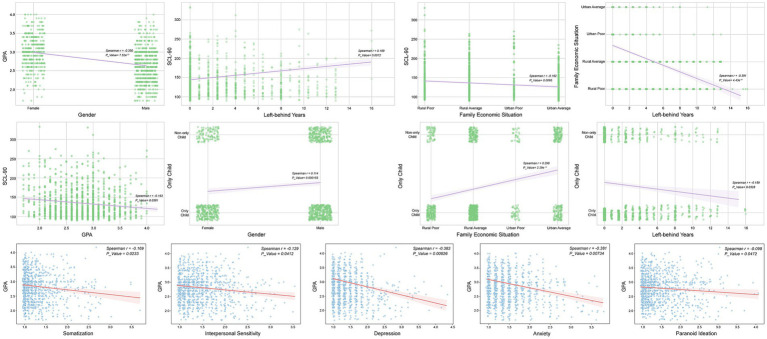
Spearman correlation analysis and visualization of psychological health, GPA, and major family background factors.

To further explore the potential relationships among students’ GPA, psychological health, and family background variables, Spearman’ s rank correlation analysis was performed for all variable pairs, as presented in Figure 6. Family economic situation was negatively correlated with left-behind years (*r* = −0.391, *p* < 0.001), suggesting that students with longer left-behind experiences tended to come from families with relatively lower economic status. Family economic situation was also positively correlated with only-child (*r* = 0.298, *p* < 0.001), indicating that students from families with higher economic conditions were more likely to be non-only children. Only-child status was negatively correlated with left-behind years (*r* = −0.189, *p* = 0.0328), suggesting that a larger proportion of only children had left-behind experiences.

At the psychological level, the SCL-90 total score was associated with several variables. It was positively correlated with left-behind years (*r* = 0.189, *p* = 0.0012), suggesting that longer left-behind experiences tended to be associated with higher levels of psychological distress. Additionally, the SCL-90 total score was negatively correlated with family economic situation (*r* = −0.162, *p* = 0.0095), indicating that lower family economic status tended to be associated with higher levels of psychological distress. The SCL-90 total score was also negatively correlated with GPA (*r* = −0.163, *p* = 0.0381), suggesting that lower academic performance tended to be associated with increased psychological difficulties.

Further analysis of the SCL-90 subscales indicated that GPA showed negative associations with several psychological dimensions. Depression showed the strongest negative association with GPA (*r* = −0.383, *p* = 0.00626), followed by Anxiety (*r* = −0.381, *p* = 0.00734). Additional dimensions, including Somatization (*r* = −0.169, *p* = 0.0233), Interpersonal Sensitivity (*r* = −0.129, *p* = 0.0412), and Paranoid Ideation (*r* = −0.098, *p* = 0.0472), also exhibited negative associations with GPA. Overall, these results indicate that depressive and anxiety symptoms showed significant inverse correlations with academic achievement, whereas other psychological factors demonstrated weaker relationships.

## 4 Discussion

As global higher education transitions into the post-pandemic era, student academic performance confronts unprecedented complexities, rendering traditional assessment methods increasingly inadequate. Against this backdrop, advances in artificial intelligence have introduced novel approaches for predicting academic performance prediction. Waheed et al. (2020) constructed a deep neural network based on interaction data from UK universities’ virtual learning environment to predict students’ final course grades. The dataset incorporated multiple behavioral features, including platform access frequency, assignment completion, and forum participation activity (Waheed et al., 2020). Yousafzai et al. (2021) proposed a hierarchical deep neural network that integrated multi-source data, such as student demographics, historical grades, and classroom participation—to predict academic performance. While behavior-based prediction studies have clear value, the post-pandemic context indicates that additional important factors should be taken into account.

In fact, students’ GPA is not an isolated indicator, but rather a comprehensive manifestation resulting from the complex interplay of their mental health status and family background. COVID-19 pandemic has not only reshaped the learning models and ecosystems of higher education but also exacerbated diversity and uncertainty in students’ psychological health and social environments (Song et al., 2024). In this context, models that rely solely on cognitive measures or learning behaviors may no longer be sufficient to accurately capture students’ true academic status. Consequently, integrating multidimensional factors such as academic performance, mental health, and family background, to establish a dynamic and accurate framework for predicting students’ academic performance can not only provide precise identification of academic risks but also help uncover underlying drivers of academic outcomes, thereby informing targeted support strategies for instructors. In this study, we addressed three interrelated research questions. First, what is the association between psychological vulnerability and academic achievement in the post-pandemic era? Second, are family background factors, such as family economic status, only-child status, and duration of left-behind experience, related to student psychological vulnerability? Finally, do family background factors and psychological vulnerability jointly contribute to differences in student academic outcomes through a cumulative risk pathway?

Regarding the first research question, our results suggest that somatization, interpersonal sensitivity, and paranoid ideation may be associated with differences in student GPA. Notably, anxiety and depressive symptoms were significantly associated with lower academic performance. These results suggest that psychological distress is not merely peripheral but may substantially affect academic performance. A systematic review and meta-analysis have reported a significant negative association between anxiety and academic performance, with higher anxiety levels linked to lower academic outcomes. Persistent anxiety disorders are associated with larger decrements in academic performance (Huang et al., 2025). Gosadi (2024) reported that 60% of students with average or lower GPAs exhibited abnormal levels of depression. Regular physical exercise demonstrated a significant protective effect against depression in the high-GPA subgroup (*p* < 0.05), while no such effect was observed in the low-GPA subgroup. Gosadi suggested that students with poor academic performance may need additional psychological support rather than relying solely on exercise interventions. The relationship between depression and academic performance may be bidirectional: depressive symptoms can impair academic functioning, while poor academic performance may increase the risk of subsequent depression (Gosadi, 2024). Additionally, Cen et al. (2025) showed in a cross-sectional study that somatization, interpersonal sensitivity, and paranoid ideation in students were all significantly negatively correlated with GPA; that is, with each increase in severity of these three symptom types, the probability of poor GPA increased. Meanwhile, Zhang et al. (2024) identified somatization, interpersonal sensitivity, and paranoid ideation as significant predictors of GPA. Notably, interpersonal sensitivity exhibited the second largest negative effect on GPA after anxiety, while somatization and paranoid ideation also had significant negative coefficients (Zhang et al., 2024). These observations corroborate our findings and further underscore the potential adverse effects of multidimensional psychological distress on academic performance.

Regarding the second research question, our findings revealed significant associations between family background factors and students’ psychological well-being. Specifically, students from economically disadvantaged families exhibited significantly higher SCL-90 total scores, indicating elevated levels of psychological distress. Similarly, students who experienced prolonged parental absence showed markedly higher psychological symptom severity. These patterns align with existing literature on the psychological consequences of socioeconomic disadvantage and parental separation. Dong et al. (2023) reported that freshmen from economically disadvantaged backgrounds were more likely to be classified into psychological distress or at-risk groups, suggesting that socioeconomic disadvantage is not only a risk variable in the distribution of mental health issues, but also a significant upstream factor of concentrated mental-health vulnerabilities among students. Gandarillas et al. (2024) similarly reported that family socioeconomic status significantly predicted university students’ psychological health, with lower family socioeconomic status associated with greater psychological distress and weaker coping skills. Furthermore, a longitudinal study reported that children experiencing prolonged parental absence from birth exhibited higher levels of depression, anxiety, self-harm, and suicide-related risk during adolescence or early adulthood. The study noted that the longer the duration of such parent–child separation, the more persistent and significant the damage to mental health will be. Even with later parental reunification, psychological recovery may be delayed or incomplete (Wu et al., 2023). These findings collectively suggest that family background factors operate as important antecedents of psychological vulnerability among college students, with both economic hardship and parental absence contributing to elevated mental health risks.

Regarding the third research question, our results further reveal a “cumulative risk effect”. The “cumulative risk effect” refers to an initial disadvantage of individuals or groups in a specific domain, which is progressively amplified and spread to multiple functional areas through systemic interactions, ultimately resulting in a cascading deterioration of overall performance (Colburn et al., 2025). In our study, the observed “cumulative risk effect” is reflected in family background acting as an upstream factor that disrupts mental health status and influences academic outcomes. This perspective is consistent with a 20-year longitudinal study that followed American adolescents over two decades. The study found that childhood socioeconomic disadvantage was not only associated with higher levels of psychological distress during adolescence, but also influenced the completion rate of higher education. These findings are consistent with a cumulative-life-course pathway in which early family disadvantage adversely affects adolescent psychological health, which may subsequently constrain academic attainment (Chen et al., 2024). Building upon the associations documented in our preceding analyses, these findings suggest a coherent pathway: family disadvantage elevates psychological vulnerability, which in turn is associated with lower academic achievement. Students from economically disadvantaged backgrounds or with prolonged parental absence had significantly higher risks of psychological symptoms, which were associated with lower GPA. The mechanistic interpretation of this pathway involves two complementary processes. On one hand, economic constraints experienced in peer interactions may accumulate into persistent psychological pressure. On the other hand, for long-term left-behind students, lack of parental emotional companionship may weaken secure attachment and emotion regulation capacities, thereby interfering with the development of psychological resilience. These symptoms were associated with lower GPA, forming a pathway from family disadvantage to mental-health difficulties and subsequent academic setbacks. However, this is not an irreversible predicament, as the school environment can play an important moderating role. Prior studies show that a positive school atmosphere can significantly enhance academic motivation among left-behind children by improving their learning adaptability, and teachers’ support further strengthens this process, thereby effectively mitigating the adverse effects of family disadvantages. These findings imply that schools, as a critical external environment, can provide essential psychological and academic support for left-behind students, helping them overcome the cumulative risk effect (Zhao et al., 2023). Finally, this phenomenon also echoes the socioeconomic realities of the post-pandemic era, where COVID-19 impacted the global economy, placing additional strain on many families that were already economically struggling. Consequently, some parents had to extend migrant work periods, causing students’ left-behind status to persist or worsen (Gu, 2024; Hallaert et al., 2023; Wang et al., 2024). The cumulative effect of family pressures may further exacerbate the adverse impacts on students’ mental health and academic performance.

From a methodological perspective, this study focuses on the challenges of predicting student GPA in the post-pandemic era and systematically integrates family background and psychological wellbeing into a prediction framework built on deep learning architectures with feature-gating mechanisms. Regarding model selection, we prioritized the complexity of the feature space over raw sample size. While the dataset is medium-scale, it exhibits significant “high-dimensional sparsity” and “heterogeneity,” necessitating a model capable of capturing intricate dependencies (Bulut and Arslan, 2024). Traditional machine learning approaches typically require manual dimensionality reduction, which risks losing fine-grained symptom information (Jia et al., 2022). In contrast, deep learning architecture allows for the direct processing of raw inputs, preserving the full information integrity of the 90-item scale and demographic variables. Furthermore, the models employed, such as TabTransformer, are specifically engineered for tabular data; rather than simply increasing network depth, they utilize multi-head self-attention mechanisms to model the correlation matrix between features. This inductive bias enables the model to automatically assign weights, effectively addressing the data complexity without relying solely on massive-scale samples. Our results indicate that the TabTransformer enhanced with a gating module achieved good accuracy in predicting student GPA. The improved performance of the gated TabTransformer appears to result from the synergy between two distinct but complementary internal data-processing strategies. First, the gating module conducts a dynamic importance assessment of the raw input, selecting and enhancing the most relevant features. The weighted features are then passed to the TabTransformer, which models interactions among key features and captures complex higher-order dependencies by evaluating their combined effects. Notably, we found that a lightweight gating structure improved model performance when processing complex, high-dimensional student feature sets. Previous studies have reported that gating mechanisms can enhance feature selection in deep models (Guan et al., 2025). For example, studies in medical image segmentation and multimodal learning have demonstrated that gating modules contribute significantly to performance gains by mitigating noise and enhancing feature extraction (Fiaz et al., 2024; Zhang et al., 2024). Although baseline models such as TabTransformer and AutoInt demonstrated acceptable performance, integrating gating modules led to consistent and substantive improvements across all tested architectures. This suggests that modeling these data requires not only capturing nonlinear interactions but also effectively filtering meaningful signals from the noise inherent in multi-item psychological scales and heterogeneous demographic variables (Natarajan et al., 2025). The gating module, implemented as a dynamic, data-driven feature selection layer (Xia et al., 2025), learns to up-weight salient predictors and suppress irrelevant or redundant information originating from the 90-item SCL-90 and other background variables. This adaptive filtering reduces noise interference, enabling downstream networks—whether Transformer-based or simpler MLP-ResNet architectures—to focus capacity on the most influential features. In our experiments, integrating the gating mechanism has even been observed to elevate the weakest baseline model MLP-ResNet, to a level surpassing most advanced models that do not incorporate this mechanism. This result underscores the potential usefulness and generalizability of gating as a preprocessing component for heterogeneous tabular data in educational and psychological studies.

In summary, our study constructed a GPA-level prediction framework integrating deep learning and feature selection, utilizing large-scale real-world student data from the post-pandemic period, with a specific focus on incorporating key factors such as family background and psychological assessments. The study found that students’ psychological health status and family environment, though often overlooked, have important predictive value for academic performance. The framework proposed in this study is helpful for educators to identify potential academic risks of students and enables them to provide more targeted support. Furthermore, our findings offer empirical evidence for constructing a more inclusive and supportive environment in the post-pandemic era.

However, several limitations should be acknowledged. First, the study data were derived from a single university, which may somewhat limit the external generalizability of the findings. Second, the cross-sectional design of this study reveals a significant correlation between predictors and GPA, with much of the work focused on analyzing the contemporaneous association structures among psychological vulnerability, family background, and academic achievement. Therefore, future research should incorporate multi-institutional and longitudinal datasets to clarify the causal pathways linking family background, temporal changes in psychological well-being, and academic trajectories, thereby enhancing the generalizability of the results.

## Data Availability

The original contributions presented in the study are included in the article/supplementary material, further inquiries can be directed to the corresponding author.
